# Birth Outcomes and Prenatal Exposure to Ozone, Carbon Monoxide, and Particulate Matter: Results from the Children’s Health Study

**DOI:** 10.1289/ehp.8111

**Published:** 2005-07-18

**Authors:** Muhammad T. Salam, Joshua Millstein, Yu-Fen Li, Frederick W. Lurmann, Helene G. Margolis, Frank D. Gilliland

**Affiliations:** 1 Department of Preventive Medicine, University of Southern California, Keck School of Medicine, Los Angeles, California, USA; 2 Sonoma Technology Inc., Petaluma, California, USA; 3 Air Resources Board, State of California, Sacramento, California, USA

**Keywords:** air pollution, birth weight, carbon monoxide, intrauterine growth retardation, maternal exposure, nitrogen dioxide, ozone, particulate matter

## Abstract

Exposures to ambient air pollutants have been associated with adverse birth outcomes. We investigated the effects of air pollutants on birth weight mediated by reduced fetal growth among term infants who were born in California during 1975–1987 and who participated in the Children’s Health Study. Birth certificates provided maternal reproductive history and residence location at birth. Sociodemographic factors and maternal smoking during pregnancy were collected by questionnaire. Monthly average air pollutant levels were interpolated from monitors to the ZIP code of maternal residence at childbirth. Results from linear mixed-effects regression models showed that a 12-ppb increase in 24-hr ozone averaged over the entire pregnancy was associated with 47.2 g lower birth weight [95% confidence interval (CI), 27.4–67.0 g], and this association was most robust for exposures during the second and third trimesters. A 1.4-ppm difference in first-trimester carbon monoxide exposure was associated with 21.7 g lower birth weight (95% CI, 1.1–42.3 g) and 20% increased risk of intrauterine growth retardation (95% CI, 1.0–1.4). First-trimester CO and third-trimester O_3_ exposures were associated with 20% increased risk of intrauterine growth retardation. A 20-μg/m^3^ difference in levels of particulate matter ≤ 10 μm in aerodynamic diameter (PM_10_) during the third trimester was associated with a 21.7-g lower birth weight (95% CI, 1.1–42.2 g), but this association was reduced and not significant after adjusting for O_3_. In summary, O_3_ exposure during the second and third trimesters and CO exposure during the first trimester were associated with reduced birth weight.

A growing body of evidence indicates that maternal exposures to air pollutants, including ozone, carbon monoxide, particulate matter with an aerodynamic diameter of ≤ 10 μm or ≤ 2.5 μm (PM_10_ and PM_2.5_, respectively), nitrogen dioxide, and sulfur dioxide, are associated with adverse pregnancy outcomes. A major research focus has been to investigate the effects of air pollution on birth weight, low birth weight (LBW; < 2,500 g), and intrauterine growth retardation (IUGR).

Williams et al. (1977) first reported an inverse association between air pollution (based on the ambient levels of CO, NO_2_, and O_3_) and birth weight in Los Angeles, California, in the 1970s. Subsequently, several studies examining the effects of air pollution on fetal growth have been conducted in the United States (Chen et al. 2002; Maisonet et al. 2001; Parker et al. 2005; Ritz and Yu 1999), Canada (Liu et al. 2003), the United Kingdom (Bobak et al. 2001), the Czech Republic (Bobak 2000; Bobak and Leon 1999), China (Wang et al. 1997), South Korea (Ha et al. 2001; Lee et al. 2003), Taiwan (Lin et al. 2004; Yang et al. 2003), and Brazil (Gouveia et al. 2004).

Data from studies that were conducted in term infants showed significant associations between CO, PM_10_, and PM_2.5_ and reduced birth weight (Chen et al. 2002; Gouveia et al. 2004; Ha et al. 2001; Lee et al. 2003; Maisonet et al. 2001; Parker et al. 2005; Ritz and Yu 1999; Yang et al. 2003) and IUGR (Dejmek et al. 1999; Ha et al. 2001; Maisonet et al. 2001; Parker et al. 2005; Ritz and Yu 1999). However, the trimester-specific findings from these studies are inconsistent because they showed different windows of increased risk of reduced birth weight and IUGR for each pollutant. The explanations for the variations in results remain unclear. Although studies suggest that O_3_ and NO_2_ may be associated with birth weight, these pollutants have received less attention in epidemiologic studies.

To further investigate the effects of O_3_, CO, PM_10_, and NO_2_ on reduced birth weight mediated by IUGR, we conducted a study among children who were born in southern California, a region with widely varying air pollution levels. We examined the associations between birth weight, LBW, and IUGR in term newborns and air pollution data in participants of the Children’s Health Study (CHS), a population-based study of children residing in 12 southern California communities.

## Materials and Methods

### Study population.

The elements of the CHS have been described previously (Peters et al. 1999a, 1999b). The population for this study was a subset of 6,259 participants in the CHS who were born in California between 1975 and 1987 and were recruited in four cohorts from public school classrooms from grades 4, 7, and 10 in 12 southern California communities in 1993–1996. At CHS entry, parents or guardians of each participating student provided written informed consent and completed a self-administered questionnaire, which included detailed demographic information as well as information regarding the mother’s and child’s respiratory health and exposure to different environmental factors that might contribute to the risk of reduced birth weight or could influence air pollution exposures. Race/ethnicity of the child was grouped as non-Hispanic white, Hispanic white, African American or black, Asian and Pacific Islander, and “other.” Maternal smoking during pregnancy was assigned using self-report of any smoking during pregnancy. Socioeconomic status (SES) was determined from questionnaire items related to parent’s educational level and annual family income at study entry (Table 1).

### Birth information.

Birth weight, gestational age, and other reproductive data were obtained from California birth certificates. Birth certificate information for California-born children who participated in the CHS was obtained by computerized linkage of participants with the California Department of Health Services Birth Statistical Master Files and Birth Cohort Files. Of the 6,259 cohort participants, 5,013 were born in California according to parental report, and 4,842 were matched to a birth certificate record. Because preterm birth is associated with adverse birth outcomes and our analyses were focused on the effects of air pollutants on birth weight mediated by reduced fetal growth as opposed to reduced gestational age, we restricted our analyses in children who were born at term with gestational ages between 37 and 44 weeks, consistent with the methods used by earlier researchers. This criterion resulted in the exclusion of 941 children who were born before the 37th week of gestation, and our final sample consisted of 3,901 children. Data obtained from California birth certificates included birth weight, gestational age, maternal age at birth, maternal residence ZIP code at birth, parity, months since last live birth, gestational diabetes, and marital status. The estimated date of conception was assigned using the birth date and gestational age, corrected for the average 2-week difference between the last menstrual period and conception. First trimester was defined as gestational age < 13 weeks, second trimester was 13–27 weeks, and third trimester was 28 weeks to birth. We defined LBW as birth weight < 2,500 g in term infants and defined IUGR by less than the 15th percentile of predicted birth weight based on gestational age and sex in term infants.

### Air pollution exposure assessment.

Air pollution estimates were assigned using the ZIP code of the maternal residence at birth and monthly average air pollution estimates for each ZIP code interpolated from monitoring data obtained from the U.S. Environmental Protection Agency’s (EPA) Aerometric Information Retrieval System through written request in 1999. Currently the 1999 data are available under the Air Quality System database of the U.S. EPA (U.S. EPA 2005). Temperature data were acquired from National Weather Service surface observations, the South Coast Air Quality Management District, the Bay Area Air Quality Management District, and CHS air pollution monitoring stations. Elevations above sea level were derived from 1-km horizontal resolution geophysical data from the U.S. Geological Survey’s Earth Resources Observation Systems Data Center (U.S. Geological Survey 2005). Elevations were assigned to the ZIP code centroid of the maternal birth residence. Exposure estimates were calculated for monthly average of 24-hr O_3_ (O_3[24hr]_), 1000 hr to 1800 hr O_3_ (O_3[10–6]_), NO_2_, CO, PM_10_, and temperature by spatially interpolated monthly average levels to the geometric centroid coordinates of the ZIP code boundaries for the ZIP code region of the maternal birth residences.

The interpolation used inverse-distance-squared weighting based on data from up to the three nearest monitoring stations located within 50 km of the ZIP code centroid (100 km for O_3_). Because the spatial gradients in monthly average data are smaller than hourly or daily data, the method was relative insensitive to the number of stations included in the interpolation and maximum interpolation radius. Three stations and a 50-km radius were selected because they preserved local gradients slightly better than did larger numbers of stations and greater distances. In cases where air monitoring data were available from a station located within 5 km of a ZIP code centroid, the assignments were based on the nearest station’s data rather than interpolation. For this cohort, 29% of the NO_2_ assignments and approximately 40% of the O_3_, PM_10_, and CO assignments were based on the nearest station’s data. Each mother’s exposures to O_3_, NO_2_, PM_10_, and CO during each trimester were estimated using weighted averages of the calendar month averages. Although other studies included SO_2_ exposures, it was not included in this study because ambient SO_2_ concentrations are generally low in California and are measured at too few monitoring stations to make reliable exposure assignments.

### Statistical analyses.

The effect of each air pollutant on birth weight and 95% confidence intervals (CIs) were computed by fitting linear mixed-effects models using the SAS procedure GENMOD (SAS version 8.2; SAS Institute Inc., Cary, NC). Because of the nonlinearity in the relationship of birth weight with gestational age, a cubic polynomial in gestational age was necessary to adequately capture the nonlinearity. We therefore included linear, quadratic, and cubic terms for gestational age in all final models. The models also included maternal age, months since last live birth, parity, maternal smoking during pregnancy, SES, marital status at childbirth, gestational diabetes, and child’s sex, race/ethnicity, and school grades (4th, 7th, and 10th) of the CHS cohort as fixed effects, and CHS study community as a random effect. To account for confounding by seasonal determinants of birth weight, the adjusted analyses included six terms for the basis matrix of a b-spline on Julian day of birth, with knots at days 91, 183, and 274 (De Boor 2001), because this has been suggested to be a more rigorous method of adjusting for temporal variations than adjusting for month or season of birth. We used missing indicators for missing data on maternal smoking during pregnancy, parity, SES, and race/ethnicity.

We considered the effects of pollutant exposures during the entire pregnancy and during each trimester. To illustrate the magnitude of the birth weight differences by air pollutant level, we presented the estimates scaled to the approximate interquartile ranges (IQRs) of average exposure for each pollutant. We also computed odds ratios (ORs) and 95% CIs for the association between pollutant levels and LBW and IUGR using appropriate logistic regression models that included the same adjustment variables as in the mixed-effects models. For pollutants that showed significant associations with birth weight in trimester-specific analysis, we fitted models that included average levels of those specific pollutants during specific trimesters. We fitted an additional model for O_3_ that included three terms for average levels during each of the three trimesters.

For pollutants that were correlated with temperature and altitude, we conducted sensitivity analyses adding linear terms for elevation above sea level, temperature, and season of birth to the multivariate models. We also fitted a model with indicator variables for the deciles of O_3[24hr]_ exposure averaged over the entire pregnancy and presented the difference in birth weight associated with each decile of O_3[24hr]_ exposure relative to the lowest decile. We assessed departure from linear relationships between birth weight and pollutants by examining appropriate residual plots and by comparing nested models with linear terms and categorical terms for each pollutant using partial *F*-tests. All significance tests were two sided at the 0.05 level.

## Results

The sample included almost equal proportions of males and females (Table 1). About 60% of subjects were non-Hispanic, and 28% were Hispanic whites. Average birth weight was 3,487 g, with 72 infants (1.8%) with LBW and 585 infants (15%) with IUGR. Approximately 18% of mothers smoked during pregnancy. Few mothers had gestational diabetes (*n* = 18) or gave twin births (*n* = 25).

Table 2 shows the means ± SDs of the air pollutants, temperature, elevation, and their correlations with one another. For the trimester-specific averages, we observed strong positive correlation between O_3[24hr]_ and O_3[10–6]_ with correlation coefficient (*r*) > 0.9, and moderate positive correlation between O_3_ and temperature with *r* approximately 0.6. Daily O_3[24hr]_ was also positively correlated with PM_10_ (*r* ~ 0.5) but negatively correlated with NO_2_ (*r* ~ −0.1) and CO (*r* ~ −0.3). Elevation was positively correlated with O_3_ (*r* ~ 0.3 −0.4) but negatively with CO (*r* ~ −0.3) and NO_2_ (*r* ~ −0.15). O_3_ levels in the first and second trimesters and the second and third trimesters were positively correlated (*r* = 0.31 in both instances), but levels in the first and third trimesters were negatively correlated (*r* = −0.25; data not shown).

Maternal exposure to O_3_ averaged over the entire pregnancy was associated with reduced birth weight for both the O_3[24hr]_ and the O_3[10–6]_ average metrics (Table 3). For O_3[24hr]_ levels during the entire pregnancy, mean birth weight was lower by 47.2 g (95% CI, 27.4–67.0 g) across the IQR of 12 ppb. Exposures to PM_10_, NO_2_, and CO when averaged over the entire pregnancy were not significantly associated with birth weight.

The inverse association between ambient O_3_ levels and birth weight was stronger for exposure occurring during the second and third trimester for both the O_3[24hr]_ and O_3[10–6]_ average metrics (Table 3). With each IQR increase (i.e., 16 ppb and 17 ppb in the second and the third trimesters, respectively) in average O_3[24hr]_ during the second and the third trimesters, birth weights were lower by 32.3 g (95% CI, 13.7–50.9 g) and 35.3 g (95% CI, 15.9–54.7 g), respectively. Trimester-specific analyses showed a statistically significant inverse association between CO concentrations in the first trimester and birth weight. Over an IQR in CO of 1.4 ppm, mean birth weight was lower by 21.7 g (95% CI, 1.1–42.3 g). The effects of PM_10_ were largest for the third trimester, and with each IQR increase in PM_10_ (i.e., 20 μg/m^3^), birth weight was lower by 21.7 g (95% CI, 1.1–42.2 g).

In sensitivity analyses where further adjustments were made for temperature, elevation, and season of birth, the effect estimates were somewhat reduced for the average O_3_ exposure over the entire pregnancy [Table S1 of the supplemental material (http://ehp.niehs.nih.gov/members/2005/8111/supplemental.pdf)]. However, greater birth weight deficits were observed for the second-trimester O_3_ exposures after such adjustment compared with the estimates presented in Table 3. Greater influence of these adjustment variables was observed on the CO effects on birth weight. The direction of association between CO exposures over the entire pregnancy and during the second and third trimesters changed from a positive to a negative one, and the negative association between first-trimester CO exposure and birth weight was attenuated. For PM_10_, the effect estimate for the third trimester was reduced and was no longer statistically significant. However, the second-trimester PM_10_ results became stronger and achieved borderline significance.

In two-pollutant models that examined the joint effects of air pollutants that showed significant associations in trimester-specific analyses, CO effects were stronger in a two-pollutant model that included O_3[24hr]_ and CO, and birth weight was lower by 28.6 g (95% CI, 6.9–50.4 g) per 1.4-ppm increase in ambient CO levels (Table 4). In contrast, in a two-pollutant model that included O_3[24hr]_ and PM_10_, the inverse association between PM_10_ and birth weight during the third trimester was attenuated and no longer was statistically significant. Including the three trimester-specific averages for O_3_ in one model attenuated the effect estimates for the second trimester but did not change the estimates for the third trimester.

Maternal O_3_ exposures averaged over the entire pregnancy and during the third trimester and CO exposure during the first trimester were significantly associated with increased risk of IUGR (Table 5). A 17-ppb difference in maternal O_3[24hr]_ exposure during the third trimester increased the risk of IUGR by 20% (95% CI, 1.0–1.3). Over an IQR in CO of 1.4 ppm during the first trimester, the risk of IUGR increased by 20% (95% CI, 1.0–1.4). A 17-ppb difference in the third-trimester O_3_ exposures was associated with 40% increased risk of LBW (95% CI, 1.0–1.9); however, the estimates had substantial uncertainty due to the small number of LBW newborns.

**Figure 1:**
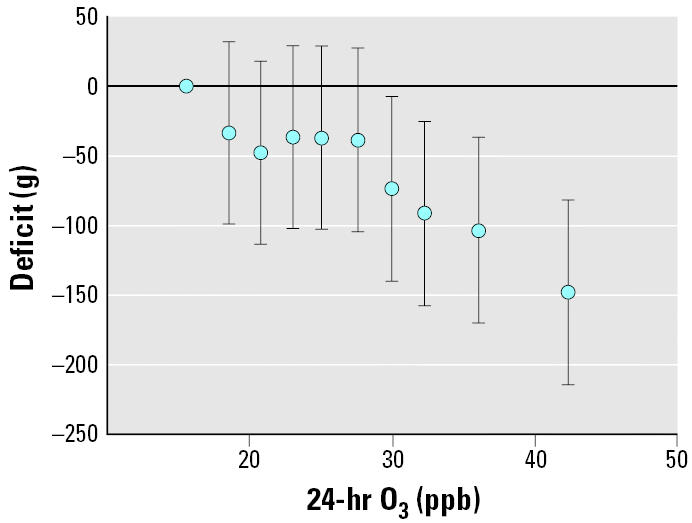
Birth weight deficit by decile of O_3[24hr]_ exposure averaged over the entire pregnancy compared with the decile group with the lowest O_3_ exposure. Deficits are plotted against the decile-group–specific median O_3_ exposure. Error bars represent 95% CIs. Indicator variables for each decile of O_3[24hr]_ exposure (except the least-exposed group) were included in a mixed model. For detailed modeling information, see “Materials and Methods.”

We also observed a dose–response relationship of birth weight with average O_3[24hr]_ that was clearest above 30-ppb exposure levels (Figure 1). Relative to the lowest decile of average O_3[24hr]_ estimates for the next five lowest deciles were approximately −40 g to −50 g, with no clear trend and with 95% confidence bounds that included zero. The highest four deciles of O_3_ exposure showed an approximately linear decrease in birth weight, and all four 95% CIs excluded zero [estimates of birth weight deficits (grams) for the four uppermost deciles of exposure, in ranked order by decile median of exposure: −73.7 (95% CI, −139.0 to −7.4); −91.6 (95% CI, −157.9 to −25.3); −103.5 (95% CI, −170.3 to −36.7); −148.3 (95% CI, −214.7 to −81.9)]. There were no consistent dose–response relationships between birth weight and CO and PM_10_ from exposure during pregnancy or by trimester (data not shown).

The crude effect estimates without any adjustment for maternal (i.e., maternal age, race, smoking status) or fetal (i.e., gestational age, sex) factors were similar to the adjusted estimates. The directions and magnitude of the effect estimates did not change in sensitivity analyses after we excluded the plural births, infants born to mothers with gestational diabetes, and subjects with missing covariate information on maternal race/ethnicity, smoking during pregnancy, parity, and SES.

## Discussion

We observed that ambient CO levels in the first trimester and O_3_ levels in the second and the third trimesters were independently associated with lower birth weight and IUGR in term infants through the mechanism of reduced fetal growth. Although PM_10_ exposure in the third trimester was associated with reduced birth weight, the PM_10_ effect was not statistically significant in a two-pollutant model that included PM_10_ and O_3_. A clear pattern of increasing deficits in birth weight with increasing levels of O_3_ was observed for 24-hr average O_3_ levels above 30 ppb, but no such trend was apparent for CO and PM_10_. NO_2_ levels were not associated with birth weight in this study. Although the differences in birth weight were small on average, those in the highest O_3_ exposure group had deficits of a magnitude equivalent (~ 150 g) to those observed after exposure to cigarette smoke.

Our finding of an inverse association between maternal CO exposure during the first trimester and birth weight and IUGR is consistent with earlier reports (Gouveia et al. 2004; Ha et al. 2001; Lee et al. 2003). Although others have observed a significant negative effect of first-trimester CO exposure on birth weight (Maisonet et al. 2001; Ritz and Yu 1999), in a study of term infants born in California in 2000, Parker et al. (2005) did not observe a significant association between CO and birth weight. This lack of a strong association between CO and birth weight in a recent California birth cohort could be due to > 3-fold reduction in average third-trimester CO exposure levels from 1989–1993 to 2000 (2.5 and 0.8 ppm, respectively) (Parker et al. 2005; Ritz and Yu 1999).

Several lines of evidence support the plausibility of a negative effect of CO exposure on birth weight. CO reduces oxygen-carrying capacity of maternal hemoglobin, which could adversely affect O_2_ delivery to fetal circulation. Because CO crosses the placental barrier (Sangalli et al. 2003) and fetal hemoglobin has greater affinity for binding CO than does adult hemoglobin, O_2_ delivery to fetal tissues is further compromised (Di Cera et al. 1989). The resultant tissue hypoxia has the potential to reduce fetal growth.

The robust inverse association of O_3_ concentrations with reduced birth weight is consistent with the early study of Williams et al. (1977) conducted in Los Angeles, California, in the 1970s. The present study found smaller deficits in birth weight across the range of O_3_ concentrations than those observed in the study conducted by Williams et al., likely because they included children who were born preterm, and the levels of O_3_ and other pollutants in the 1970s were much higher than levels during our study period. The 150-g deficit we observed in the highest decile of exposure in our study is of the same order of magnitude as the 314-g weight reduction in babies from areas with high O_3_ levels compared with those living in the least-polluted areas, as was observed by Williams et al. (1977).

Of the more recent published reports that assessed the role of different ambient air pollutants on birth weight, a limited number assessed the effects of O_3_ levels. Data from these studies suggested an inverse association between current ambient O_3_ levels and birth weight (Chen et al. 2002; Gouveia et al. 2004; Ha et al. 2001). Chen et al. (2002) observed a borderline significant inverse association between ambient O_3_ levels and birth weight in term infants born in Washoe County, Nevada, between 1991 and 1999. Mean 8-hr O_3_ levels in Washoe County were substantially lower compared with the levels observed in this study (27.8 vs. 50.6 ppb). In Seoul, South Korea, with a median O_3_ level of 22.4 ppb, Ha et al. (2001) also observed a significant association between levels of O_3_ in the third trimester and LBW. Our results are consistent with the findings from these studies. In the study of term infants in Los Angeles, conducted by Ritz and Yu (1999), CO effects were the focus of the research, and associations of O_3_ with birth weight were not reported. In addition, in this study, we observed significant reductions in birth weight with O_3[24hr]_ concentrations ≥ 30 ppb. This may provide an explanation for not observing any association between O_3_ and birth weight in areas with lower ambient O_3_ levels. However, interpreting this observation as evidence for a threshold is not justified without further investigation.

In addition to epidemiologic reports, experimental studies in animal models also support a role of O_3_ in reduced birth weight and suggest that the effect could be mediated through modulation of maternal inflammatory processes. In rats, neutrophilic inflammation was proportional to O_3_ dose, and pregnant rats were more susceptible to acute pulmonary inflammation from O_3_ than were virgin rats (Gunnison and Hatch 1999). The increased susceptibility during pregnancy arises from higher O_3_ doses to the respiratory epithelial lining fluid (RELF) due to higher alveolar ventilation produced by increased tidal volumes and decreased ascorbic acid levels in the RELF. Kavlock et al. (1979) reported that mid and late gestational exposure to O_3_ increased embryo toxicity in rats with evidence for a reduction in weight gain 6 days after birth. Bignami et al. (1994) observed reduced postnatal weight gain in mice whose mothers were exposed to 1.2 ppm O_3_ over days 7–17 of gestation. Because pregnancy duration in mice is about 3 weeks (Thibodeaux et al. 2003), 7–17 days of gestation would correspond to the second and the third trimesters in mice. In a second study by this group, mice exposed prenatally to 0.6 ppm O_3_ showed a long-lasting reduction in body weight (Dell’Omo et al. 1995).

Similar experimental studies of exposures cannot be conducted in pregnant women; however, in healthy human volunteers, effects of O_3_ exposures on biomarkers have been observed, including increased peripheral neutrophil and lower ascorbic acid levels 6 hr after 0.2 ppm O_3_ exposure for 2 hr (Mudway et al. 1999). Because pregnant women have higher alveolar ventilation than do nonpregnant women (Barclay 1997), the level of exposures during pregnancy is likely to be greater, and similar inflammatory responses could be more pronounced in pregnant women. Furthermore, inflammation due to O_3_ results in the release of lipid peroxidation products and inflammatory cytokines into circulation (Hemmingsen et al. 1999; Larini and Bocci 2005). This could adversely affect placental circulation and function and can affect fetal growth. Further research is needed to define the mechanisms of O_3_ on the maternal–fetal unit.

Our estimates of about a 22-g decrease in birth weight per 20-μg/m^3^ increase in PM_10_ during the third trimester was comparable with the 11-g decrease in birth weight for a 10-μg/m^3^ increase in PM_10_ levels, as observed by Chen et al. (2002). However, after adjustment for O_3_, the estimate was reduced by about 50% and was not statistically significant. Further investigation of the relationship of PM_10_ and birth outcomes is needed, especially in the context of exposures to ambient levels of O_3_ and PM_10_.

Although a South Korean research group observed a significant negative effect of first-trimester NO_2_ exposure on birth weight in two studies (Ha et al. 2001; Lee et al. 2003) where the study population of the first study was part of the second study, other researchers did not observe any significant association between NO_2_ and birth weight (Gouveia et al. 2004; Lin et al. 2004; Liu et al. 2003). Moreover, there was a high correlation between NO_2_, CO, and total suspended particles or PM_10_ in the studies conducted in South Korea, and it is not clear whether NO_2_ had an independent negative effect in these studies.

Although third-trimester O_3_ exposure was positively associated with LBW, we did not observe any significant association between any of the measured pollutants and LBW in our sample for exposures averaged over the entire pregnancy or for exposures during the first and second trimesters. One reason for observing significant associations between air pollutants and birth weight on a continuous scale but not on a categorical scale could be due to the low prevalence (i.e., < 2%) of LBW. Because a set cut-point of 2,500 g is used to define LBW, and the prevalence of LBW in term infants in developed countries is low, the use of LBW may not be ideal for assessing the impact of air pollution on birth weight in developed countries.

In contrast with earlier studies, the present study had information on maternal smoking and SES to adjust for potential confounding by these factors. Because maternal smoking during pregnancy may affect the associations between air pollutants and birth weight, and we did not have enough power to detect any significant effect modification, we restricted our analysis to mothers who did not smoke during their pregnancy with the index child and observed similar results (data not shown).

The findings from this study must be interpreted in light of the methods used to assign exposure during the period of pregnancy. Air pollution exposures were assigned based on the centroid of the ZIP code of maternal residence at childbirth and may not reflect the actual exposure levels for the duration of the entire pregnancy. Studies have shown that about 20–25% of pregnant women change their residences during pregnancy (Khoury et al. 1988; Shaw and Malcoe 1992). However, pregnant women most often move to a different house in the same locality (Fell et al. 2004), probably because of the proximity to their workplaces, health care providers, and schools for those with older children. Residential address from birth certificates most often reflects maternal location during the last trimester (Schulman et al. 1993). This may have minimized the measurement error in exposure assessment during the third trimester, and as such, the third-trimester–specific results are likely to have less error than do the other trimester-specific results. We also lacked information on maternal employment location and commuting patterns. Because the resultant misclassification in exposure assignment is not likely to be differential, it may have attenuated the risk estimates. Exposure misclassification may explain the null findings for NO_2_, but this would not account for the deficits in birth weight that we observed from exposures to the other pollutants. Because we did not have direct measures of maternal exposures to indoor and occupational pollutants during pregnancy, we could not address the effects of these exposures on the birth outcomes.

Another source for error in the air pollution estimates may be the distance between maternal residence and the air monitoring stations used to assign exposure. The proportion of data on O_3_, PM_10_, NO_2_, and CO interpolated from monitoring stations < 5 km from the maternal birth residence ranged from 29.2 to 41.1%, whereas 51.2–61.3% were between 5 and 25 km, and the remaining 2.8–9.6% were interpolated from stations between 25 and 50 km for PM_10_, NO_2_, and CO and between 25 and 100 km for O_3_ [Table S2 of the supplemental material (http://ehp.niehs.nih.gov/members/2005/8111/supplemental.pdf)]. We conducted sensitivity analyses to assess the influence of distance of the monitoring stations on the associations between air pollutants and birth outcomes excluding women living farther from these monitors, and the results showed little change (data not shown). Furthermore, a recent study in California showed high correlation (*r* ~ 0.9) of air pollutant levels between monitors located within 5 miles (i.e., ~ 8 km) from the maternal residence and those located at the county level (Basu et al. 2004), suggesting that the ZIP-code–based air pollution data could be a good proxy for exposure levels at the residences and in the region surrounding residences where subjects spend most of their time.

Although measurement errors of pollutants in single-pollutant models are likely to attenuate the magnitude of birth weight deficits associated with air pollutants, the findings from two-pollutant models as well as those adjusted for temperature and elevation should be evaluated with some caution given the correlation structure between these environmental factors. In two-pollutant models where levels of pollutants are correlated and the measurement error in one pollutant is larger than the other, Zeger et al. (2000) showed that the effects of the relatively poorly measured pollutant are decreased and could introduce positive bias in the effect of the better measured pollutant in the presence of strong negative correlation between the measurement errors of the two pollutants. We would expect attenuation in the CO effect estimate in the first trimester in a two-pollutant model that included O_3_, because the measurement error in CO was possibly larger than O_3_ and the pollutants were negatively correlated (*r* ~ 0.3). However, the CO effect estimates remained robust in a two-pollutant model, suggesting an independent CO effect on birth weight in the first trimester. PM_10_ and O_3_, on the other hand, were positively correlated in the third trimester (*r* ~ 0.5), and the effect of PM_10_ was reduced about 2-fold in the two-pollutant model. Because a strong negative correlation between the measurement errors in PM_10_ and O_3_ seems unlikely, the independent PM_10_ effects may be smaller.

Measures of maternal nutrition (i.e., prepregnancy weight-for-height, gestational weight gain, and intake of nutrients) were not available to assess the potential effects of these factors on the associations observed in this study. Although maternal nutrition may be a determinant of birth weight in the developing world, Kramer (1987) did not observe any significant role of maternal nutrition in LBW in developed countries. Also, earlier literature has shown that maternal race/ethnicity (Cohen et al. 2001), maternal education (Kramer et al. 2000), and maternal age (Haiek and Lederman 1989) are associated with maternal nutrition. Because we adjusted for race, maternal education, and maternal age and found little evidence for confounding in our analyses, we may have indirectly adjusted for the nutritional differences during pregnancy. Therefore, maternal nutrition is unlikely to confound the associations of air pollutants with birth weight in this study.

We also considered the possibility of bias from the potential effects of air pollutants on preterm births. In California, birth certificate data for birth weight have been found to be valid and reliable (Von Behren and Reynolds 2003). However, the data on gestational age based on maternal recall of last menstrual period may be less accurate. We may have included some preterm births in our analysis, but because our outcome of interest was reduction in mean birth weight, our results are not likely to be biased by the effects of air pollutants on gestational age at birth.

In conclusion, we observed an association between lower birth weight and IUGR with O_3_ concentrations. The second- and third-trimester O_3_ levels were most strongly associated with deficits in birth weight. In addition, CO levels in the first trimester were associated with about a 22-g reduction in birth weight over an IQR of 1.4 ppm. These findings suggest that ambient CO in the first trimester and O_3_ in the second and third trimesters are determinants of birth weight and IUGR. Because exposures to the levels of ambient air pollutants observed in this study are common, and fetal growth is an important determinant for childhood and adult morbidity and mortality, our findings are likely to have important public health and regulatory implications.

## Supplementary Material

supplemental material

## Figures and Tables

**Table 1:** Characteristics of study subjects (*n* = 3,901)^a^ born at term in California between 1975 and 1987.

Characteristic	No. (%)
Gestational age (days)^b^	281.7 ± 10.3
Birth weight (g)^b^	3,486.7 ± 489.0
Sex (male)	1,888 (48.4)
LBW (< 2,500 g)	72 (1.8)
Term infants with IUGR	585 (15.0)
Maternal smoking during pregnancy	717 (18.8)
Unmarried at childbirth	632 (16.2)
Maternal gestational diabetes	18 (0.5)
Race/ethnicity	
Non-Hispanic white	2,315 (59.7)
Hispanic white	1,096 (28.3)
African American/black	183 (4.7)
Asian/Pacific Islander	100 (2.6)
Other	184 (4.7)
Maternal age at childbirth (years)	
< 20	356 (9.1)
20–22	607 (15.6)
23–29	1,917 (49.1)
30–32	550 (14.1)
33–35	298 (7.6)
≥36	173 (4.4)
Parity	
1	1,630 (41.9)
2	1,299 (33.4)
3	603 (15.5)
4	225 (5.8)
5	68 (1.8)
6–9	64 (1.6)
Months since last live birth	
0 (plural births)	25 (1.3)
10–25	537 (27.4)
26–36	464 (23.7)
36–57	446 (22.8)
≥58	485 (24.8)
SES based on parent’s/guardian’s education and annual household income	
Less than high school education or annual income < $15,000	838 (22.0)
Completed high school with annual income ≥$15,000	655 (17.2)
Some college education with annual income ≥$15,000	1,421 (37.2)
Completed 4-year college with annual income ≥$15,000	310 (8.1)
Graduate-level education or annual family income ≥$100,000	593 (15.5)

a Data are presented as *n* (%) unless otherwise specified. Subjects with missing data were not used to calculate the percentages.

b Data presented as mean ± SD.

**Table 2:** Correlations for average exposures within each trimester of pregnancy.

		Correlations						
Exposure	Mean ± SD	O_3[10–6]_	O_3[24hr]_	PM_10_	NO_2_	CO	Temperature	Elevation
Entire pregnancy								
O_3[10–6]_ (ppb)	50.6 ± 17.5	1	0.85	0.54	0.26	0.00	0.41	0.43
O_3[24hr]_ (ppb)	27.3 ± 8.7		1	0.20	−0.10	−0.27	0.17	0.60
PM_10_ (μg/m^3^)	45.8 ± 12.9			1	0.55	0.41	0.49	−0.02
NO_2_ (ppb)	36.1 ± 15.4				1	0.69	0.54	−0.16
CO (ppm)	1.8 ± 0.9					1	0.32	−0.35
Temperature (°F)	61.2 ± 4.2						1	−0.19
Elevation (m)	219.1 ± 263.5							1
First trimester								
O_3[10–6]_ (ppb)	51.0 ± 28.2	1	0.92	0.54	0.12	−0.17	0.69	0.28
O_3[24hr]_ (ppb)	27.5 ± 14.1		1	0.34	−0.11	−0.32	0.60	0.38
PM_10_ (μg/m^3^)	46.6 ± 15.9			1	0.48	0.29	0.47	0.00
NO_2_ (ppb)	36.6 ± 16.9				1	0.68	0.20	−0.13
CO (ppm)	1.8 ± 1.1					1	−0.07	−0.29
Temperature (°F)	61.9 ± 7.3						1	−0.12
Second trimester								
O_3[10–6]_ (ppb)	49.9 ± 25.5	1	0.91	0.50	0.10	−0.20	0.67	0.27
O_3[24hr]_ (ppb)	27.0 ± 12.8		1	0.27	−0.15	−0.37	0.57	0.38
PM_10_ (μg/m^3^)	45.4 ± 14.8			1	0.53	0.35	0.48	−0.04
NO_2_ (ppb)	36.2 ± 16.9				1	0.72	0.21	−0.15
CO (ppm)	1.8 ± 1.1					1	−0.04	−0.30
Temperature (°F)	61.5 ± 6.8						1	−0.14
Third trimester								
O_3[10–6]_ (ppb)	51.1 ± 27.1	1	0.91	0.52	0.11	−0.22	0.72	0.29
O_3[24hr]_ (ppb)	27.5 ± 13.3		1	0.31	−0.14	−0.40	0.63	0.40
PM_10_ (μg/m^3^)	45.4 ± 15.5			1	0.52	0.37	0.48	−0.01
NO_2_ (ppb)	35.5 ± 16.6				1	0.71	0.18	−0.15
CO (ppm)	1.8 ± 1.1					1	−0.05	−0.30
Temperature (°F)	61.7 ± 7.1						1	−0.08

**Table 3:** Effects of air pollutants on birth weight from single-pollutant models.^a^

	IQR	Birth weight [g (95% CI)]^b^	*p*-Value
Entire pregnancy			
O_3[10–6]_	26 ppb	−49.9 (−72.0 to −27.8)	< 0.001
O_3[24hr]_	12 ppb	−47.2 (−67.0 to −27.4)	< 0.001
PM_10_	18 μg/m^3^	−19.9 (−43.6 to 3.8)	0.10
NO_2_	25 ppb	−7.2 (−34.7 to 20.4)	0.61
CO	1.2 ppm	2.2 (−20.1 to 24.4)	0.85
First trimester			
O_3[10–6]_	33 ppb	−5.7 (−23.2 to 11.8)	0.52
O_3[24hr]_	17 ppb	−10.4 (−28.6 to 7.7)	0.26
PM_10_	20 μg/m^3^	−3.0 (−22.7 to 16.7)	0.76
NO_2_	25 ppb	−15.3 (−39.7 to 9.2)	0.22
CO	1.4 ppm	−21.7 (−42.3 to −1.1)	0.04
Second trimester			
O_3[10–6]_	29 ppb	−25.7 (−42.7 to −8.7)	0.003
O_3[24hr]_	16 ppb	−32.1 (−50.7 to −13.4)	< 0.001
PM_10_	19 μg/m^3^	−15.7 (−36.1 to 4.7)	0.13
NO_2_	25 ppb	1.9 (−23.1 to 26.9)	0.88
CO	1.4 ppm	11.3 (−9.7 to 32.3)	0.29
Third trimester			
O_3[10–6]_	33 ppb	−36.7 (−54.9 to −18.5)	< 0.001
O_3[24hr]_	17 ppb	−35.2 (−54.6 to −15.8)	< 0.001
PM_10_	20 μg/m^3^	−21.7 (−42.2 to −1.1)	0.04
NO_2_	25 ppb	−6.1 (−31.0 to 18.9)	0.63
CO	1.3 ppm	11.8 (−8.4 to 32.1)	0.25

a For detailed modeling information, see “Materials and Methods.”

b Minus sign denotes reduction in mean birth weight.

**Table 4:** Effects of air pollutants on birth weight estimated from multipollutant models.^a^

	IQR	Birth weight [g (95% CI)]^b^	*p*-Value
Model 1: first trimester			
O_3[24hr]_	17 ppb	−17.8 (−37.3 to 1.6)	0.07
CO	1.4 ppm	−28.6 (−50.4 to −6.9)	0.01
Model 2: third trimester			
O_3[24hr]_	17 ppb	−31.6 (−52.2 to −11.0)	0.003
PM_10_	20 μg/m^3^	−10.8 (−31.8 to 10.2)	0.31
Model 3: all trimesters			
O_3[24hr]_ (first trimester)	17 ppb	−18.6 (−39.0 to 1.7)	0.07
O_3[24hr]_ (second trimester)	16 ppb	−17.5 (−38.6 to 3.7)	0.11
O_3[24hr]_ (third trimester)	17 ppb	−36.9 (−58.9 to −15.0)	< 0.001

a For detailed modeling information, see “Materials and Methods.”

b Minus sign denotes reduction in mean birth weight.

**Table 5:** Effects of air pollutants on LBW and IUGR from single-pollutant models.

		IUGR		LBW	
	IQR	OR (95% CI)^a^	*p*-Value	OR (95% CI)^a^	*p*-Value
Entire pregnancy					
O_3[10–6]_	26 ppb	1.2 (1.0 to 1.5)	0.02	1.5 (0.9 to 2.3)	0.10
O_3[24hr]_	12 ppb	1.2 (1.0 to 1.4)	0.01	1.3 (0.9 to 1.8)	0.18
PM_10_	18 μg/m^3^	1.1 (0.9 to 1.3)	0.49	1.3 (0.8 to 2.2)	0.22
NO_2_	25 ppb	1.1 (0.9 to 1.3)	0.51	0.8 (0.4 to 1.4)	0.44
CO	1.2 ppm	1.0 (0.9 to 1.2)	0.62	0.8 (0.6 to 1.3)	0.41
First trimester					
O_3[10–6]_	33 ppb	1.0 (0.9 to 1.1)	0.92	1.0 (0.7 to 1.3)	0.92
O_3[24hr]_	17 ppb	1.0 (0.9 to 1.2)	0.72	1.0 (0.7 to 1.3)	0.87
PM_10_	20 μg/m^3^	1.0 (0.9 to 1.2)	0.94	1.0 (0.7 to 1.5)	0.85
NO_2_	25 ppb	1.2 (1.0 to 1.4)	0.07	0.9 (0.5 to 1.5)	0.67
CO	1.4 ppm	1.2 (1.0 to 1.4)	0.01	1.0 (0.7 to 1.5)	0.90
Second trimester					
O_3[10–6]_	29 ppb	1.1 (1.0 to 1.2)	0.18	1.1 (0.8 to 1.5)	0.47
O_3[24hr]_	16 ppb	1.1 (1.0 to 1.2)	0.10	1.1 (0.8 to 1.5)	0.65
PM_10_	19 μg/m^3^	1.0 (0.9 to 1.2)	0.74	1.2 (0.8 to 1.7)	0.34
NO_2_	25 ppb	1.0 (0.8 to 1.2)	0.94	1.0 (0.6 to 1.6)	0.90
CO	1.4 ppm	1.0 (0.9 to 1.1)	0.72	0.9 (0.6 to 1.3)	0.66
Third trimester					
O_3[10–6]_	33 ppb	1.2 (1.0 to 1.3)	0.01	1.4 (1.1 to 1.9)	0.02
O_3[24hr]_	17 ppb	1.2 (1.0 to 1.3)	0.02	1.4 (1.0 to 1.9)	0.03
PM_10_	20 μg/m^3^	1.1 (0.9 to 1.3)	0.29	1.3 (0.9 to 1.9)	0.13
NO_2_	25 ppb	1.0 (0.8 to 1.2)	0.93	0.6 (0.4 to 1.1)	0.11
CO	1.3 ppm	1.0 (0.8 to 1.1)	0.51	0.7 (0.5 to 1.1)	0.09

a For detailed modeling information, see “Materials and Methods.”
