# Supplementary material for: Birth Outcomes and Prenatal Exposure to Ozone, Carbon Monoxide, and Particulate Matter: Results from the Children’s Health Study
Source: Environ Health Perspect. 2005 Jul 18;113(11):1638–44. doi: 10.1289/ehp.8111 (PMC1310931; doi:10.1289/ehp.8111)
Supplement: supplemental material [file ehp0113-001638s1.pdf]

Birth Outcomes and Prenatal Exposure to Ozone, Carbon Monoxide, and Particulate Matter: Results from the Children's Health Study

Muhammad T. Salam[1], Joshua Millstein[1], Yu-Fen Li[1], Frederick W. Lurmann[2], Helene G. Margolis[3], Frank D. Gilliland[1]

[1]Department of Preventive Medicine, University of Southern California, Keck School of Medicine, Los Angeles, California

[2] Sonoma Technology, Inc., Petaluma, California

[3] Air Resources Board, State of California, Sacramento, California

Supplemental Material

Table S1: Effects of air pollutants on birth weight from single pollutant models further adjusted for temperature, elevation, and season of birth[a].

	Interquartile range	Birth weight[b]	(95% CI)	*p*-value
Entire Pregnancy				
$O_{3\ [10\text{-}6]}$	26 ppb	-38.4	(-68.9, -8.0)	0.01
$O_{3\ [24hour]}$	12 ppb	-43.0	(-71.1, -14.8)	0.003
PM_{10}	18 µg/m^3	-11.7	(-38.1, 14.7)	0.39
NO_2	25 ppb	-6.7	(-37.7, 24.3)	0.67
CO	1.2 ppm	-8.4	(-35.3, 18.4)	0.54
First Trimester				
$O_{3\ [10\text{-}6]}$	33 ppb	-19.1	(-48.5, 10.4)	0.20
$O_{3\ [24hour]}$	17 ppb	-32.4	(-63.9, -1.0)	0.04
PM_{10}	20 µg/m^3	-1.4	(-24.3, 21.5)	0.90
NO_2	25 ppb	0.4	(-27.6, 28.3)	0.98
CO	1.4 ppm	-15.5	(-43.7, 12.7)	0.28
Second Trimester				
$O_{3\ [10\text{-}6]}$	29 ppb	-32.5	(-60.6, -4.5)	0.02
$O_{3\ [24hour]}$	16 ppb	-37.9	(-69.4, -6.4)	0.02
PM_{10}	19 µg/m^3	-23.8	(-47.7, 0.0)	0.05
NO_2	25 ppb	-9.2	(-37.3, 18.9)	0.52
CO	1.4 ppm	-5.8	(-33.7, 22.0)	0.68
Third Trimester				
$O_{3\ [10\text{-}6]}$	33 ppb	-35.5	(-66.4, -4.6)	0.02
$O_{3\ [24hour]}$	17 ppb	-28.3	(-63.6, 7.0)	0.12
PM_{10}	20 µg/m^3	-17.9	(-42.2, 6.4)	0.15
NO_2	25 ppb	-18.9	(-47.4, 9.6)	0.20
CO	1.3 ppm	-8.4	(-35.4, 18.7)	0.55

[a]Fixed covariates included maternal age, months since last live birth, parity, maternal smoking during pregnancy, socioeconomic status, marital status at childbirth, maternal diabetes, gestational age with quadratic and cubic terms, child's sex, race/ethnicity, school grade, temperature, elevation, and season of birth. Study community of subject at study entry was included as a random effect. The adjusted analyses also included 6 terms for the basis matrix of a

b-spline on Julian day of birth. The b-spline included 3 knots at days 91, 183, and 274. First trimester was defined as gestational age 0-12 weeks, second trimester was 13-27 weeks, and third trimester was 28-delivery.

[b]Minus sign denotes reduction in mean birth weight.

Table S2. Distance from Maternal Birth Residence to Nearest Monitoring Station.

	(percent of exposure data)		
	<5km	5-25km	>25km[a]
$O_{3\ [10\text{-}6]}$	39.9	52.6	7.6
$O_{3\ [24hour]}$	40.6	52.0	7.4
PM_{10}	41.0	56.1	2.8
NO_2	29.2	61.3	9.6
CO	41.1	51.2	7.7

[a]The maximum distance from a maternal birth residence to a monitoring station was 50 km for all exposure components except ozone, which was 100 km.
